# Plasma Generator with Dielectric Rim and FSS Electrode for Enhanced RCS Reduction Effect

**DOI:** 10.3390/s21248486

**Published:** 2021-12-20

**Authors:** Taejoo Oh, Changseok Cho, Wookhyun Ahn, Jong-Gwan Yook, Jangjae Lee, Shinjae You, Jinwoo Yim, Jungje Ha, Gihun Bae, Heung-Cheol You, Yongshik Lee

**Affiliations:** 1 Department of Electrical and Electronic Engineering, Yonsei University, Seoul 03722, Korea; taejoo.oh@yonsei.ac.kr (T.O.); changseok.cho@yonsei.ac.kr (C.C.); wookhyun.ahn@yonsei.ac.kr (W.A.); jgyook@yonsei.ac.kr (J.-G.Y.); 2 Department of Physics, Chungnam National University, Daejeon 34134, Korea; leejj3800@cnu.ac.kr (J.L.); sjyou@cnu.ac.kr (S.Y.); 3 Agency for Defense Development (ADD), Daejeon 34186, Korea; jwyim@add.re.kr (J.Y.); jungjeha@add.re.kr (J.H.); gbae@add.re.kr (G.B.); addyhc@add.re.kr (H.-C.Y.)

**Keywords:** dielectric-barrier-discharge, FSS, plasma, radar cross section, X-band

## Abstract

In this study, a method was experimentally verified for further reducing the radar cross-section (RCS) of a two-dimensional planar target by using a dielectric rim in a dielectric barrier discharge (DBD) plasma generator using a frequency selective surface (FSS) as an electrode. By designing the frequency selective surface such that the passbands of the radar signal match, it is possible to minimize the effect of the conductor electrode, in order to maximize the RCS reduction effect due to the plasma. By designing the FSS to be independent of the polarization, the effect of RCS reduction can be insensitive to the polarization of the incoming wave. Furthermore, by introducing a dielectric rim between the FSS electrode and the target, an additional RCS reduction effect is achieved. By fabricating the proposed plasma generator, an RCS reduction effect of up to 6.4 dB in X-band was experimentally verified.

## 1. Introduction

Advances in radar technology have led to the development of low observable (LO) technology, which can increase the survivability of an aircraft. Among various LO technologies, electromagnetic stealth technology that reduces the radar cross-section (RCS) has been extensively studied. RCS is a measure of the ability of a target to reflect incoming radar signals [1]. It is the area intercepting that amount of power which, if radiated isotropically, produces the same power received by the radar. It is given by:

(1) $$\sigma =\underset{r\to \infty }{\lim}4\pi r^{2}\frac{{\left|E_{s}\right|}^{2}}{{\left|E_{i}\right|}^{2}},$$

where *E_s_* and *E_i_* is the scattered and incident electric field, and *r* is the distance between the target and the radar. There are some available methods to reduce RCS. For instance, the reduction of the signal incident on an aircraft from the radar can be achieved by modifying the structure of the aircraft to induce scattered reflection. Another method is to absorb electromagnetic waves incident on an aircraft using a radar absorbing material or radar absorbing structure [1,2,3,4]. However, these methods adversely affect the dynamics of an aircraft by deforming its body and structure, and entail continuous maintenance costs due to the short durability of the lossy body during aircraft operation.

The stealth technique using plasma has recently attracted attention as a method for reducing RCS. The dielectric constant of plasma is often modeled by the plasma frequency and the collision frequency via the Drude model. The real part of the plasma dielectric constant is 1 or less, and the imaginary part has a relatively large value such that the loss is higher than that of other materials. Therefore, extensive research has been conducted based on the suitability of plasma in detection technology [5,6,7,8,9].

A previous study confirmed the stealth effect by applying an inductively coupled plasma generator to the S-shaped inlet of an aircraft and analyzing the reduction in RCS according to the discharge power applied to the plasma generator through measurements [5]. As the discharge power increased, the RCS reduction effect increased; up to 25 dB was reduced at 6 GHz for the incident power level of 500 W. However, the inductively coupled plasma generator requires a separate cooling device for stable operation due to the high temperatures during plasma generation. In addition, there is a limit to the increase of the plasma generation area; accordingly, the volume and weight increase, being unsuitable for use in an aircraft.

The dielectric barrier discharge (DBD) generator is relatively thin, does not require a separate cooling device, and can generate plasma over a large area, thus being easy to apply to large areas such as an aircraft. Another study analyzed the effect of reducing plasma RCS according to the change of the plasma driving frequency by designing a parallel capacitor plate type DBD generator, and it was confirmed through an experiment that the maximum decrease in RCS according to the presence or absence of plasma was 3.1 dB [9]. However, these plasma detection studies resulted in relatively small RCS reductions compared to other methods.

In this study, we propose a frequency selective surface (FSS) electrode dielectric barrier structure plasma generator applied with a dielectric rim. An FSS is a surface that provides a response selective to a specific frequency band. It can pass a signal of certain frequencies and reflect others, or vice versa. Among various types, the cross dipole FSS is one of the most popular types of FSS, which is relatively simple to design [10]. By designing an electrode based on cross-dipole FSS with a passband of 10 GHz, the effect of the conductor electrode is minimized and the amount of contact between the X-band signal and plasma is maximized. In addition, it is possible to maintain the effect of RCS reduction regardless of the polarization of the incoming signal, by designing the FSS to be independent of the polarization. Further reduction in the RCS is achieved by adding a dielectric rim to scatter the radar signal. The designed generator confirmed the possibility of operation as a plasma generator in a flow environment through manufacturing and discharge experiments, and up to 6.4 dB of RCS reduction effect was verified in X-band through RCS measurements.

## 2. Dielectric Barrier Discharge (DBD) Plasma Generator with Frequency Selective Surface (FSS) Electrode

**Figure 1:**
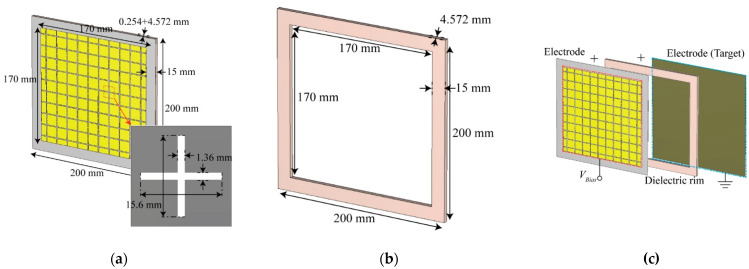
Components of the proposed dielectric barrier discharge (DBD) structure plasma generator: (**a**) frequency selective surface (FSS) electrode, (**b**) dielectric rim, (**c**) three-dimensional (3D) layer structure.

Figure 1a shows the FSS electrode of the plasma generator proposed in this study. The top electrode is an FSS based on cross dipoles, designed at 10 GHz. When biased, the plasma is generated on top of the FSS. Thus, when an X-band signal is incident, it comes into contact with the plasma first, and then is transmitted through the FSS to reach the target. The electrode was designed by printing copper on a Rogers RO4350B substrate with a thickness of 0.254 mm and a dielectric constant of 3.66. Using an FR-4 dielectric rim with a nominal thickness of 4.572 mm [11], the gap between the FSS electrode and the conductor target is maintained the same as the thickness of the dielectric. Thus, the DBD generator efficiently generates plasma over a large area. By making the area of the dielectric larger than the area of copper, additional space is maintained on the top, bottom, left, and right directions. The extra dielectric in the perimeter of the FSS layer provides a dielectric barrier to the fringing fields, which prevents the generation of unwanted arc plasma. Moreover, the designed electrode has a symmetrical structure, enabling constant RCS reduction regardless of radar polarization.

Figure 1b shows the structure of the proposed dielectric rim. The dielectric consists of three FR-4 1.524 mm thin films with a dielectric constant of 4.3, and is placed between the electrode and the ground plane to maintain the spacing of 4.572 mm between the electrode and the target. In addition, the radar signal passing through the plasma generator electrode is scattered in different directions by the dielectric rim, further reducing monostatic RCS. The proposed generator is manufactured by bonding electrodes, dielectrics, and targets as shown in Figure 1c.

**Figure 2:**
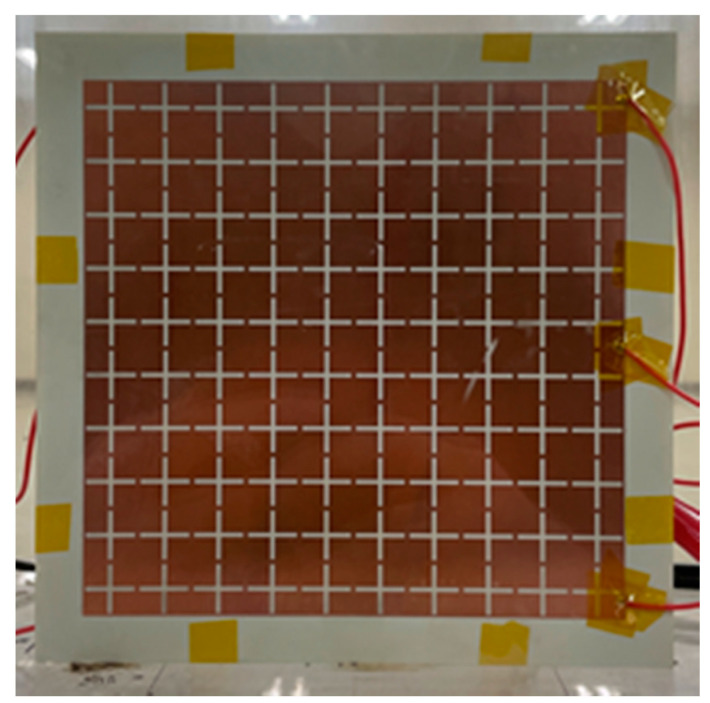
Fabricated DBD structure plasma generator.

Figure 2 shows the final fabrication of the proposed DBD generator with an FSS electrode. Plasma is generated when a voltage higher than the breakdown voltage is applied between the electrodes and the ground. In this study, plasma was generated at 0.3 atm, the atmospheric pressure at 30,000 ft (approximately 9.1 km), which is the altitude where an aircraft operates.

Finally, the top view fabricated generator is shown in Figure 2. The ground plane is a 20 × 20 cm^2^ copper flat plate on the backside, which is used as a target when measuring RCS.

**Figure 3:**
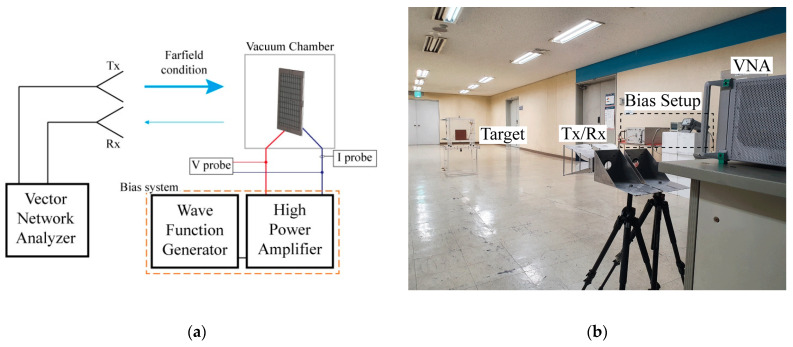
Monostatic radar cross-section (RCS) experimental environment: (**a**) block diagram, (**b**) photograph.

Figure 3 shows the experimental setup for monostatic RCS measurement using the proposed DBD plasma generator. After placing the fabricated generator in an acrylic chamber with a volume of 400 × 300 × 400 mm^3^, the experiment was conducted by generating plasma with the atmospheric pressure in the chamber adjusted to 0.3 atm using a vacuum pump.

In the case of the proposed generator, the gap between the electrode and ground (target) was 4.572 mm, which is maintained by the dielectric rim between the two; thus, the breakdown voltage of the proposed plasma generator was approximately 8 kV peak-to-peak at 0.3 atm according to Paschen’s law [8,12]. Therefore, plasma was stably generated when a higher voltage was applied. In this study, a signal was generated using Keysight’s 3350B function generator. The generated signal was amplified to several kV voltage using Trek’s 10/40A high voltage amplifier and applied to the DBD generator.

The experiment was conducted by connecting Anritsu’s MS4640B vector network analyzer and two horn antennas with a gain of 15–18 dBi in X-bands, one to transmit and the other to receive the signal reflected from the target. Throughout the experiment, the distance between the antenna and the target was maintained at approximately 5 m, which satisfies the far-field condition. For the measured signal, only the reflected signal of the target was selected through time gating, and the RCS was calculated through post-processing. In this experiment, the RCS of the target can be calculated by the following equation [13].

(2) $$\sigma_{target}=\sigma_{ref}\frac{{\left|\eta_{target}-\eta_{back}\right|}^{2}}{{\left|\eta_{ref}-\eta_{back}\right|}^{2}}$$

In (2), $\sigma_{ref}$ is the RCS of the reference target whose RCS is known. Also, $\eta_{target}$ is the measured reflection coefficient of the target, $\eta_{ref}$ is measured reflection coefficient of the reference target, and $\eta_{back}$ is reflection coefficient of background without the target or the reference target. The subtraction is to remove the background noise. By comparing the difference in RCS before and after plasma generation, the degree of RCS reduction by plasma was obtained.

## 3. Experimental Results and Analysis 

The plasma characteristics generated from the DBD generator considerably vary depending on the frequency and voltage of the bias signal as well as the surrounding gas and pressure [9]. In this study, the experiment was conducted by changing the bias signal to identify the conditions for the optimal plasma generation with the naked eye.

**Figure 4:**
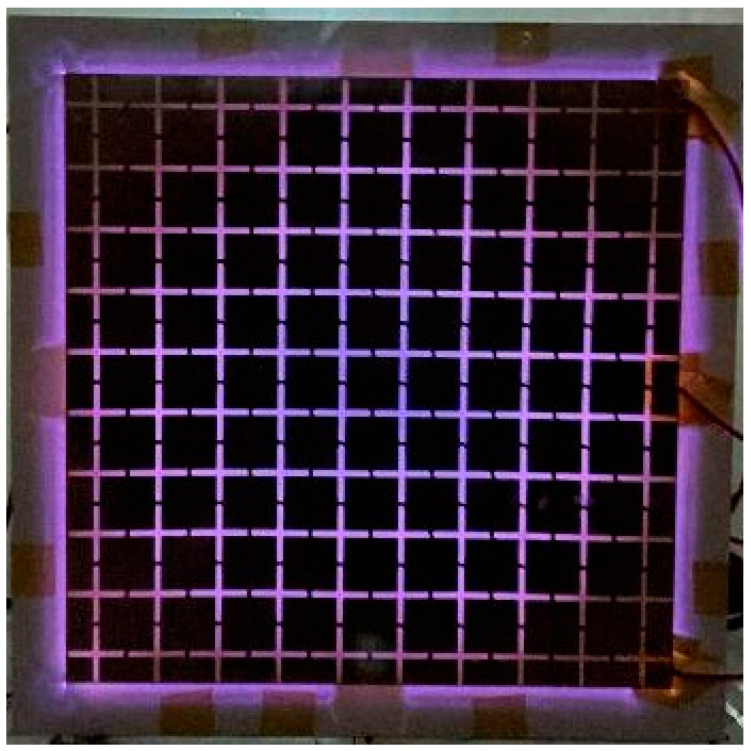
Plasma generation form of the fabricated generator.

**Figure 5:**
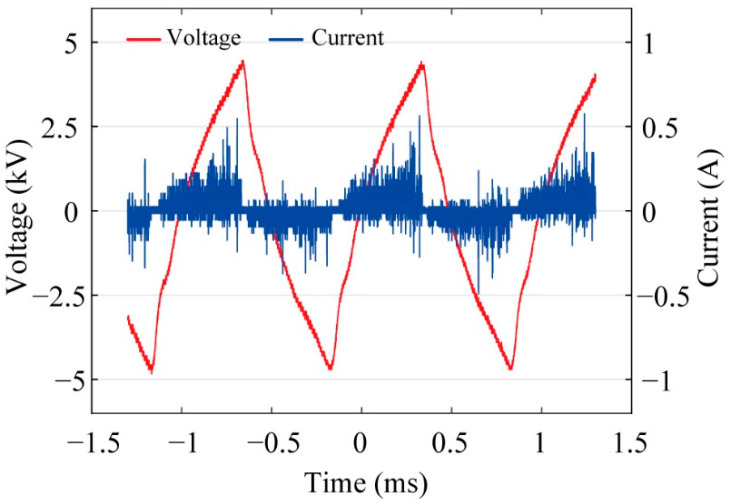
Voltage and current value of optimal plasma.

Figure 4 shows the optimal plasma generation form of the fabricated DBD generator. At this time, the plasma was generated at a driving frequency of 1 kHz and an applied peak-to-peak voltage of 8 kV. With a further increase in the applied bias voltage to peak-to-peak voltage of 10 kV, a stable plasma was generated throughout the generator. The discharge voltage and current of the plasma generator measured is shown in Figure 5. The measured peak-to-peak voltage was 8.9 kV, which was slightly reduced compared to the applied voltage because of the loss resulting from the use of a high voltage amplifier. The calculated average power consumption for generation of the plasma is 80.5 W.

**Figure 6:**
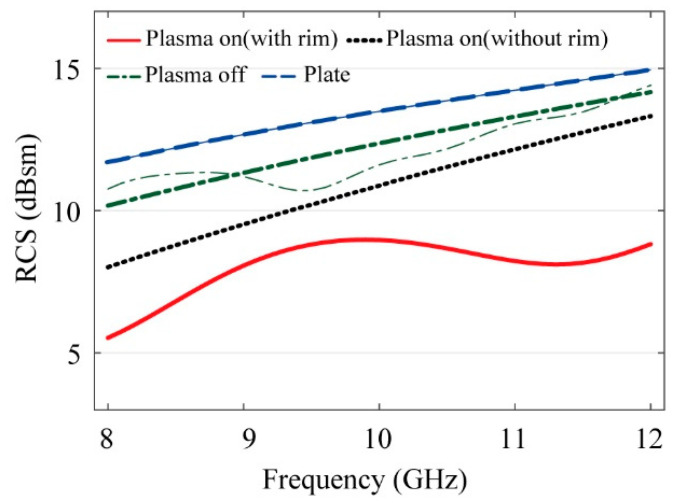
Measured (thick) and simulated (thin) RCS results: target (20 × 20 cm^2^ copper plate), target with DBD (plasma off), target with DBD (plasma on with and without dielectric rim). Simulated results are those of the target.

Figure 6 shows measured RCS of the target, which is the 200 × 200 mm^2^ copper plate with and without the FSS-based DBD plasma generator. The figure also compares the measured RCS, with and without the plasma, as well as with and without the dielectric rim. For comparison, the simulated results for the 200 × 200 mm^2^ copper plate from CST is also provided. The results confirmed that the measured RCS of the copper plate was in good agreement with the simulation. In the case of the generator with the DBD electrode and dielectric attached to the copper plate, the signal was scattered from the antenna due to the additional structure. Therefore, the RCS of the DBD generator was reduced compared to that of the copper plate. Although the RCS of the DBD generator was measured through time gating in an indoor environment, the results were similar to the simulation results. The driving frequency was 1 kHz, the applied peak-to-peak voltage was 10 kV, and the overall RCS in the X-band decreased when plasma was generated. It was verified through experiments in which the decrease in RCS due to the addition of the dielectric rim was greater compared to the case without the dielectric rim.

**Figure 7:**
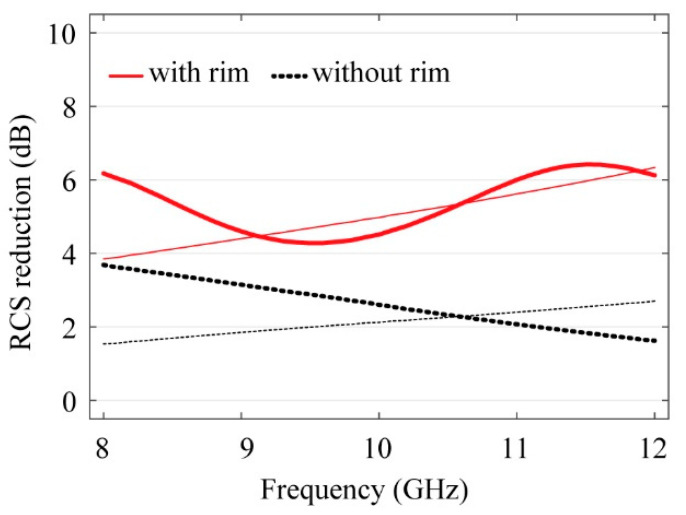
Comparison of RCS reduction effect of proposed DBD: with and without dielectric rim. Measured (thick) and simulated (thin) results based on the Drude model using CST [14].

Figure 7 shows the measured RCS of the 200 × 200 mm^2^ copper plate and when the proposed DBD generator is attached to this plate. The figure also shows the changes in the RCS when the plasma is generated, with and without the dielectric rim. Results reveal that by attaching the FSS-based DBD generator to the plate, RCS is reduced by as much as 1.5 dB. When a bias voltage is applied and the plasma is generated, the RCS is reduced further by as much as 2.2 dB. Finally, with a dielectric rim, an additional of 2.4 dB of RCS reduction effect is measured. The RCS of the copper plate decreased over the entire X-band, with an average reduction of 5.4 dB. The maximum reduction was 6.4 dB at 11.5 GHz, and the minimum reduction was 4.3 dB at 9.5 GHz.

For comparison, Figure 7 also shows the full-wave simulated results obtained thru CST [11]. Simulations are performed based on the Drude model in Equation (3), which is a popular model that describes its interaction between plasma and electromagnetic waves [8,9,12,15,16].

(3) $$\epsilon \;\left(\omega \right)=\epsilon_{\infty }-\frac{\omega_{p}^{2}}{\omega^{2}+j\omega \nu }.$$

Here, *ϵ*_∞_ is the permittivity when the frequency is infinite, ω  is the angular frequency, $\omega_{p}$ is the plasma frequency, and $\omega_{c}$ is the collision frequency. In the vacuum state when plasma is absent, $\omega_{p}$ = 0 and the permittivity is 1; thus, Equation (3) is simplified to *ϵ*_∞_ = 1. The plasma can be considered as a lossy medium with the electromagnetic properties in Equation (3) to obtain the simulated RCS. From these simulations, the effective plasma parameters are obtained such that the simulated results fit the measured results. 

Assuming 684 GHz of collision frequency at 0.3 atm [8], the extracted plasma frequency is 240 Grad/s, which gives the best match between the measured and simulated RCS results. This corresponds to an electron density of 1.81 × 10^13^ cm^−3^. This relatively high electron density is not the actual electron density provided the RCS reduction effect, but rather the effective density that takes into account the effect of not only the plasma, but also the scattering by the rim as well as various other associated loss parameters. Comparison between the experimental and fitted curves in Figure 7 shows reasonable agreement between the two. The discrepancy is relatively large for the results without the rim, indicating that there may be different mechanism of RCS reduction that cannot be modelled exactly with the Drude model. This remains to be investigated. In Table 1, the plasma parameters used in the modeling of Figure 6 are summarized.

## 4. Conclusions

In this study, we proposed the DBD plasma generator, based on FSS electrodes, applicable to a flow environment. The proposed plasma generator uses 10 GHz passband FSS to design an electrode, such that the signal of the X-band radar passes through the electrode as much as possible. This minimizes the effect of the conductor plasma, which in turn maximizes the effect of lossy plasma. Further reduction in the RCS is achieved by placing a dielectric rim between the electrode and the target, since it induces more scattering of the incoming wave. Experimental results reveal that the monostatic RCS of a 200 × 200 mm^2^ copper plate is reduced substantially over the entire X-band, with a maximum reduction of 6.4 dB at 11.5 GHz. Conclusively, the experimental and simulation results were consistent, proving that the proposed plasma generator is effective in reducing RCS. The proposed system can be embedded at hot spots, the places that contribute the most to the RCS of the entire structure, to maximize the effect under practical conditions. For future challenges, optimization of a plasma generator that can be applied to a 3D structure rather than a simple two-dimensional area should be studied.

## Figures and Tables

**Table 1:** Summary of extracted plasma parameters.

	With Dielectric Rim	Without Dielectric Rim
ω_p_	240 Grad/s	150 Grad/s
ν_p_	684 GHz	684 GHz
*n*_e_	1.81 × 10^13^ cm^−3^	7.07 × 10 ^12^ cm^−3^
